# Complete Genome Sequence of *Vibrio anguillarum* Phage CHOED Successfully Used for Phage Therapy in Aquaculture

**DOI:** 10.1128/genomeA.00091-14

**Published:** 2014-07-10

**Authors:** Jaime Romero, Gastón Higuera, Felipe Gajardo, Daniel Castillo, Mathias Middleboe, Katherine García, Carolina Ramírez, Romilio T. Espejo

**Affiliations:** a Laboratorio de Biotecnología, Instituto de Nutrición y Tecnología de los Alimentos (INTA), Universidad de Chile, Santiago, Chile; b Department of Biology, University of Copenhagen, Helsingør, Denmark

## Abstract

*Vibrio anguillarum* phage CHOED was isolated from Chilean mussels. It is a virulent phage showing effective inhibition of *V. anguillarum*. CHOED has potential in phage therapy, because it can protect fish from vibriosis in fish farms. Here, we announce the completely sequenced genome of *V. anguillarum* phage CHOED.

## GENOME ANNOUNCEMENT

*Vibrio anguillarum* (also known as *Listonella anguillarum*) is a marine Gram-negative bacterium that is the etiologic agent of vibriosis, a fatal hemorrhagic septicemia that affects >50 fresh- and saltwater fish species, including species of economic importance to the aquaculture industry (salmonids, flatfish, seabass, and seabream). Antibiotics are still the main therapeutic tools for controlling bacterial diseases in aquaculture. However, their use is becoming restricted due to their environmental impact and the emergence of resistant bacteria (1). Furthermore, antibiotics are usually delivered in the feed, and this may affect the normal microbiota of fish (2). Hence, alternative methods of treatment are needed, and phage therapy is a potential alternative approach for controlling pathogenic bacteria in aquaculture. In this context, Higuera et al. (3) showed the successful use of CHOED phage as a prophylactic tool protecting fish from vibriosis in farming conditions, as when CHOED phage was added, 100% of the salmon survived the challenge.

*V. anguillarum* phage CHOED was isolated from Chilean mussels, harvested in the south of Chile. Electron microscopy indicated that the CHOED phage has an icosahedral head around 50 nm in diameter, and no evident tail was observed; hence, it belongs to the family *Podoviridae*. The CHOED phage was concentrated by ultracentrifugation in a CsCl gradient, and the precipitated phage was suspended in synthetic seawater. Genomic DNA was extracted by the alkaline lysis method (3). The whole-genome sequencing of *V. anguillarum* phage CHOED was performed using a 454 GS Junior system.

The data generated from the genomic library provided 20,019 reads, with an average read length of 439.57 nucleotides. The sequences were assembled in a single contig, with a median coverage of 47.99×. The quality filtering and control of the data set were performed using Fastx-Toolkit (http://hannonlab.cshl.edu/fastx_toolkit/) and FastQC (http://www.bioinformatics.babraham.ac.uk/projects/fastqc/), respectively. The assembly of the reads was performed using MIRA assembler (4), and the result was a single chromosome of 66,912 bp, with 43.65% G+C content. The ends were not determined; hence, the terminal repeats are unknown. The annotation of the genome was made using Rapid Annotations using Subsystems Technology (RAST) (5) and Blast2GO (6), resulting in 94 protein-coding genes, nine of which were predicted as membrane protein-coding genes using PSORTb (7). Analysis using tRNAScan-SE (8) revealed two tRNA genes, one for Asn and one for Ser codons. This genome contains functional genes related to DNA phage synthesis (DNA helicase and DNA-directed DNA polymerase) and nucleic acid processing (ATP-dependent DNA ligase, RNA ligase, 5′-3′ exonuclease, DNA-directed RNA polymerase, homing endonuclease, and terminase) and related to phage structure (p22 coat protein and portal protein) and tail structure for host interaction (tail fiber repeat family protein). These functional genes are organized in the CHOED genome, representing a T7-like genome organization, with early genes, DNA metabolism genes, and viral structure and assembly genes.

### Nucleotide sequence accession number.

The complete sequence of the *V. anguillarum* phage CHOED genome can be accessed under the GenBank accession no. KJ192399.
